# Exploring the Enigma of a Urachal Mucinous Cystic Tumor of Low Malignant Potential (MCTLMP): A Case Report and Literature Review

**DOI:** 10.7759/cureus.78562

**Published:** 2025-02-05

**Authors:** Klaas De Corte, Ali Ramadhan

**Affiliations:** 1 Department of Pathology, Antwerp University Hospital, Edegem, BEL

**Keywords:** adenocarcinoma, histopathology, immunohistochemistry staining, kras mutations, low malignant potential, mucinous cystic tumor, pseudomyxoma peritonei, urachal neoplasms, urachus, vesicourachal diverticulum

## Abstract

Mucinous urachal neoplasms are rare and can present as benign cystadenomas, mucinous cystic tumors of low malignant potential (MCTLMPs), or invasive adenocarcinomas. MCTLMPs occupy a borderline category characterized by mild-to-moderate atypia and papillary/villous architecture without stromal invasion. Here, we describe a 54-year-old male patient who presented with persistent dysuria and was found to have a vesicourachal diverticulum. Surgical management involved a robot-assisted partial cystectomy with complete excision of the urachal remnant. Histopathologic examination revealed a multiloculated cystic lesion lined by mucin-producing epithelium, confirming an MCTLMP; immunohistochemical staining demonstrated intestinal differentiation (CK20+/CK7-/CDX2+) and absence of invasive features. p53 demonstrated wild-type expression, indicating no significant molecular alterations. The early postoperative course was uneventful, with no complications such as infection or urinary leakage. At the one-year follow-up, the patient experienced persistent urinary urgency, suprapubic discomfort, and scrotal pain, indicative of lower urinary tract dysfunction secondary to surgical intervention. No recurrence has been observed. This case underscores the importance of accurate histopathological evaluation, complete surgical resection, and vigilant long-term surveillance to prevent progression of urachal mucinous tumors. Further research into molecular markers may refine prognostication and guide future therapeutic strategies for these rare neoplasms.

## Introduction

The urachus is an embryologic remnant of the allantois that connects the bladder dome to the umbilicus. Although urachal neoplasms are rare, comprising only 0.2-0.7% of bladder cancers, they can be diagnostically challenging and clinically significant due to nonspecific symptoms and potential for late detection [1-4]. Mucinous neoplasms of the urachus mirror those found in the appendix and ovary, ranging from benign cystadenomas to mucinous cystic tumors of low malignant potential (MCTLMPs) and invasive mucinous adenocarcinomas [5-12].

MCTLMPs are particularly uncommon borderline lesions, displaying mild-to-moderate atypia with a complex papillary or villous architecture but without stromal invasion. Accurate classification of MCTLMPs is essential because clinical management differs substantially across the spectrum of urachal mucinous tumors. While benign lesions require only simple excision, higher-grade tumors may need additional therapy to forestall complications such as pseudomyxoma peritonei (PMP) [3,9,10]. Furthermore, molecular studies have shown the presence of KRAS mutations and other molecular alterations in mucinous urachal neoplasms, which may aid in understanding tumor behavior and guiding future therapeutic interventions [11].

Rare complications, such as PMP, can arise when mucinous tumors rupture or invade, leading to widespread peritoneal involvement. While PMP is more commonly associated with appendiceal neoplasms, it has also been reported in cases of urachal mucinous tumors, emphasizing the importance of early diagnosis and complete surgical excision to prevent progression [12].

Due to the rarity of these tumors, the literature is limited to case reports and small series, underscoring the need for further investigation to establish standardized diagnostic criteria and management protocols [13,14]. Atypical presentations, such as metastatic spread to unusual sites, further complicate diagnosis and highlight the aggressive potential of some urachal tumors [15].

Here, we present a case of a mucinous cystic tumor of the urachus (low malignant potential) in a 54-year-old man who presented with persistent dysuria and was found to have a vesicourachal diverticulum on imaging. We also provide a literature review highlighting the key histopathological and immunophenotypic features of MCTLMPs, emphasizing the importance of complete surgical excision and ongoing follow-up.

## Case presentation

Clinical presentation

In February 2024, a 54-year-old man presented to the emergency department with four days of progressive dysuria and mild lower abdominal discomfort. He also reported a burning sensation during micturition and a band-like pain in the lower abdomen. He had a long-standing congenital renal anomaly (crossed fused renal ectopia) noted in his past medical records and a presumed urachal cyst diagnosed incidentally several years prior.

On initial evaluation, his vital signs revealed a fever of up to 38.5°C, heart rate of 69 beats/min, blood pressure of 111/77 mmHg, respiratory rate of 15 breaths/min, and oxygen saturation of 95% on room air. Laboratory tests showed leukocytosis (white blood cell count of 13.8 × 10^9/L with neutrophil predominance; reference range: 4.2 - 10.3 × 10⁹/L) and an elevated C-reactive protein (CRP) of 137 mg/L (upper limit <10 mg/L), suggesting an active inflammatory process. Urinalysis was negative for pyuria or bacteriuria.

Imaging workup

**Figure 1:**
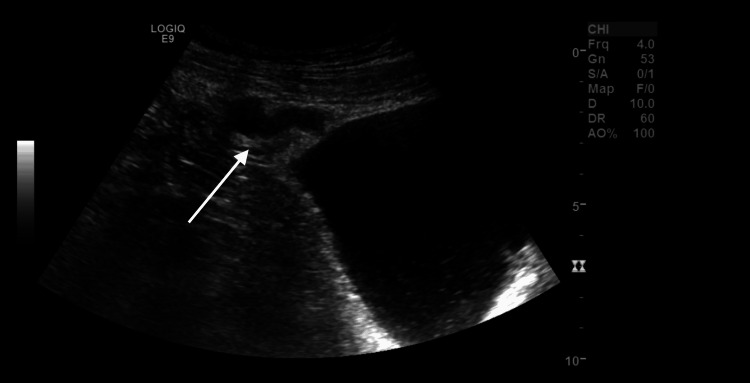
Ultrasound image demonstrating a tubular, cystic lesion at the bladder dome (arrow), consistent with a vesicourachal diverticulum.

Ultrasound of the abdomen (Figure 1) revealed a tubular cystic structure (approximately 3.5 cm in diameter) at the anterosuperior aspect of the bladder dome, suspicious for a vesicourachal diverticulum. The patient’s kidneys demonstrated a fused, crossed ectopic configuration (“L-shaped” or “horseshoe-like” kidney), with no evidence of hydronephrosis. There was some sludge in the gallbladder but no signs of cholecystitis.

**Figure 2:**
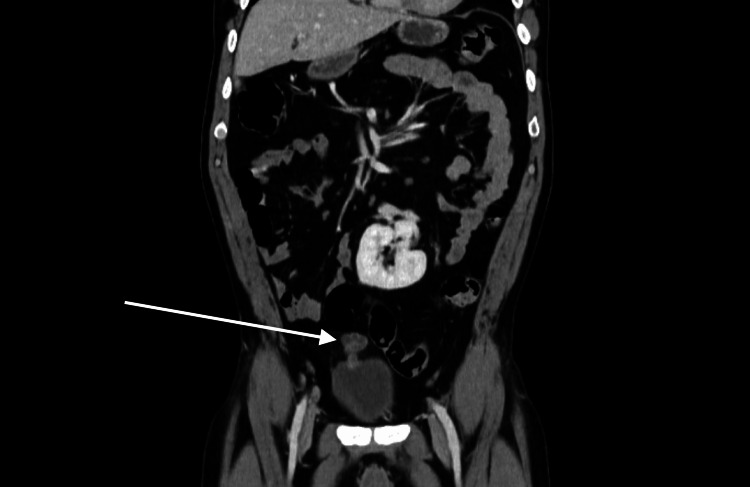
Contrast-enhanced CT of the abdomen showing a well-defined cystic lesion (arrow) at the anterosuperior aspect of the bladder with mild surrounding inflammatory changes.

Computed tomography (CT) of the abdomen with contrast (Figure 2) confirmed the vesicourachal diverticulum with enhancing walls and mild surrounding fat stranding. No signs of distant metastases were noted.

Given his persistent urinary symptoms, the likely infection of the diverticulum was managed with oral ciprofloxacin (500 mg twice daily for 14 days). On outpatient follow-up, he continued to have dysuria and a painful sensation in the left testicle, prompting further evaluation by urology. Cystoscopic examination revealed a small orifice in the dome region corresponding to the diverticulum.

Surgical intervention

Owing to recurrent symptoms and the risk of ongoing infection or potential neoplastic change within the diverticulum, a robot-assisted partial cystectomy with complete excision of the urachal remnant was performed. Intraoperatively, the diverticulum was identified at the bladder dome and carefully dissected from surrounding tissue. No gross evidence of invasive disease was noted. A Foley catheter was left in place postoperatively.

Pathological findings

Gross Examination

**Figure 3:**
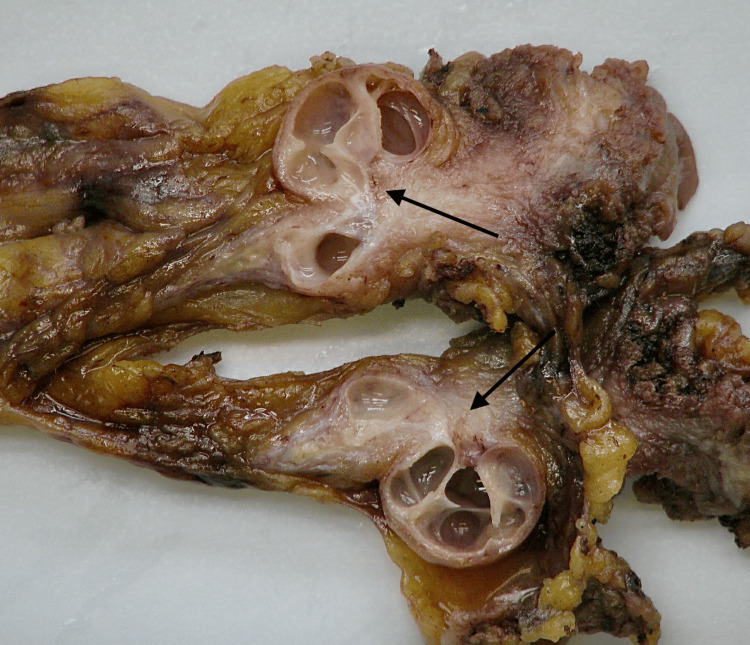
Gross photograph of the bisected specimen highlighting cystic areas (arrows).

The specimen measured 17 cm in length and up to 4 cm at its widest point. Sectioning revealed a multiloculated cystic lesion filled with viscous, mucinous material. The cyst walls were heterogeneous, with smooth translucent areas and regions of nodular thickening. The proximal end tapered into a fibrous tract toward the bladder, and no necrosis, hemorrhage, or solid masses were observed (Figure 3).

Histopathology

**Figure 4:**
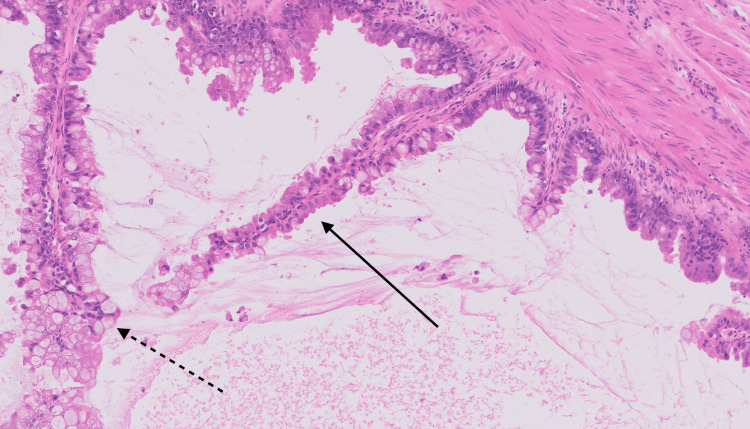
High-power hematoxylin and eosin (H&E) image revealing papillary/villous (solid arrow) with goblet cells (dotted arrow).

**Figure 5:**
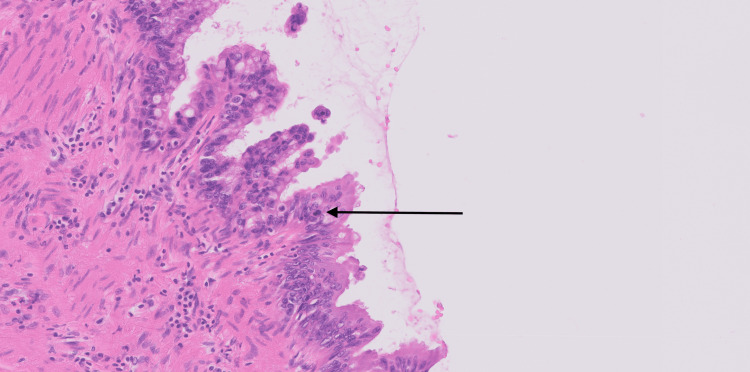
High-power H&E demonstrating mild-to-moderate atypia with occasional mitotic figures (arrow).

Microscopic examination revealed a multiloculated cystic lesion lined by mucin-producing epithelium. The lining displayed a papillary and villous architecture. In some areas, a urothelial lining was present, while other regions showed intestinal-type epithelium with goblet cells (Figure 4). The intestinal-type epithelium exhibited nuclear stratification and mild-to-moderate atypia. Occasional tufting and elongated papillary structures were observed. Stromal invasion was not identified. Mitotic figures were present but rare, indicating low proliferative activity (Figure 5).

Immunohistochemistry

**Figure 6:**
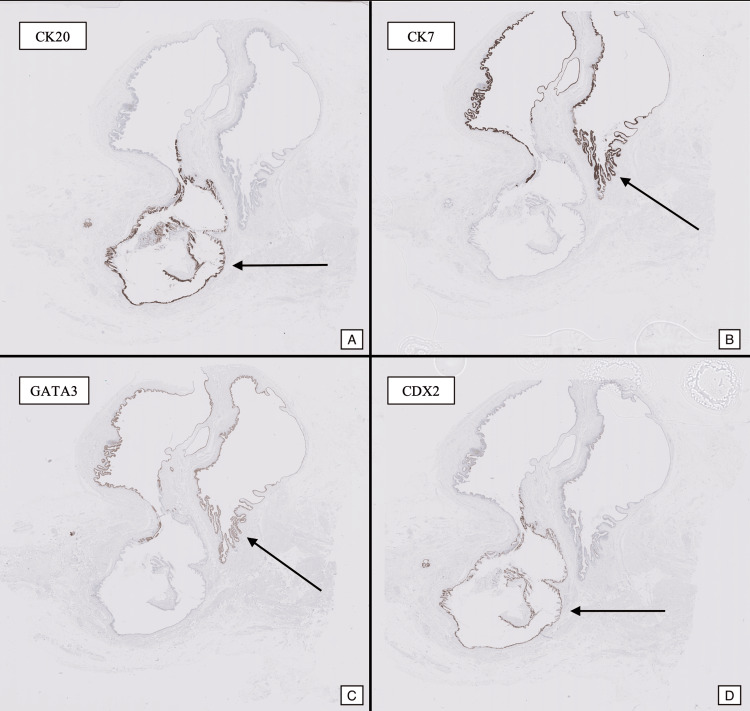
Immunohistochemical staining for CK20/CDX2 and CK7/GATA highlighting intestinal and mucinous differentiation respectively (arrows).

CK20 was expressed in the mucinous and intestinal-type areas. CDX2 was also positive in these areas, supporting intestinal differentiation. GATA3 showed focal positivity in the urothelial lining, indicating transitional differentiation. CK7 was negative in the mucinous epithelium but positive in scattered urothelial cells (Figure 6). p53 demonstrated wild-type expression, suggesting no significant molecular alterations. MUC5AC was positive in the mucinous epithelium, while MUC2 was positive in the intestinal-type epithelium; both were negative in the urothelial areas. SATB2 showed strong positivity in the mucinous and intestinal areas, confirming intestinal differentiation.

Diagnosis and Interpretation

The findings were consistent with an MCTLMP arising in the urachus. While some areas showed atypical epithelial features, these were confined to the epithelium without stromal invasion, excluding a diagnosis of invasive carcinoma. The lesion was completely excised, and surgical margins were uninvolved, indicating a favorable outcome.

Postoperative course

The early postoperative phase was uncomplicated, with minimal urine leakage resolving under continued catheter drainage. At the six-week follow-up, the patient reported no dysuria or abdominal pain, and cystoscopy demonstrated no residual or recurrent disease. However, at the one-year follow-up, he developed mild urinary urgency, suprapubic discomfort, and scrotal pain. These symptoms were attributed to lower urinary tract dysfunction, likely related to the bladder surgery. These symptoms were effectively managed with NSAIDs and a combination of solifenacin and tamsulosin, leading to significant symptomatic improvement. However, long-term sequelae, including persistent lower urinary tract symptoms, remain a potential concern necessitating continued surveillance.

## Discussion

Literature review and case context

Mucinous neoplasms of the urachus represent a rare spectrum of disease, ranging from benign cystadenomas to invasive adenocarcinomas. MCTLMPs occupy an intermediate position, displaying mild-to-moderate epithelial atypia with papillary/villous architecture, mucin production, and no stromal invasion [5-8,11]. Proper classification is critical, as management and outcomes vary significantly across the spectrum. Benign lesions can be managed with simple excision, while more advanced or invasive lesions require comprehensive surgical intervention and may require systemic therapy [2,6].

The presented case demonstrates the clinical and pathological features of an MCTLMP. Imaging revealed a tubular cystic lesion, consistent with previous reports describing vesicourachal diverticula associated with urachal mucinous tumors [9,13]. The histopathological findings of papillary/villous structures and mild-to-moderate atypia without invasion confirmed the diagnosis of MCTLMPs. This aligns with the classification system proposed by Amin et al. (2014), which emphasizes the absence of stromal invasion as the defining feature of these borderline lesions [6].

While this case highlights favorable outcomes following surgical resection, it also underscores the importance of long-term monitoring. Even in cases with complete excision, lower urinary tract dysfunction or other sequelae may arise, necessitating prompt evaluation and management [12].

Molecular insights and prognostic implications

Molecular studies on urachal mucinous neoplasms are limited but evolving. KRAS mutations, commonly identified in mucinous tumors of the appendix and ovary, have also been implicated in urachal tumors, suggesting shared pathways of tumorigenesis [11]. These mutations may serve as prognostic markers or therapeutic targets, particularly in advanced or recurrent disease. Similarly, biomarkers such as circulating tumor DNA and serum CEA/CA19-9 levels could aid in early detection and monitoring of residual or recurrent disease [12]. However, their clinical utility in urachal tumors remains underexplored, necessitating further research.

Emerging molecular studies, such as those by Reis et al. (2018), also highlight the need for a deeper understanding of the genetic underpinnings of urachal neoplasms [11]. Incorporating molecular profiling into routine practice could refine diagnosis and stratify patients for tailored therapeutic approaches. In this case, the lack of invasive features and the wild-type expression of p53 suggest a favorable prognosis, as aberrant or mutated p53 expression is often associated with more aggressive behavior and poor outcomes in urachal adenocarcinomas. Wild-type p53 expression, as observed in this case, indicates preserved tumor suppressor function, which correlates with reduced malignant potential and a lower risk of progression [6,11].

Clinical management and follow-up

Management of urachal mucinous neoplasms depends on the tumor's histological and molecular characteristics. For benign lesions such as cystadenomas, simple excision is curative, with negligible recurrence rates [2,6]. In contrast, MCTLMPs require complete surgical excision, including removal of the urachal tract and any associated diverticula, to prevent complications like progression to adenocarcinoma and PMP [12]. The presented case reinforces this approach, as complete resection resulted in an excellent short-term outcome.

For invasive adenocarcinomas, more extensive resection with lymphadenectomy may be necessary, particularly in the presence of nodal metastasis or advanced disease. Systemic therapies, including fluorouracil-based chemotherapy or targeted therapies, are often employed for metastatic cases, although standardized regimens are lacking [8,10]. Early diagnosis and appropriate surgical management remain the most critical factors for improving prognosis, especially in aggressive cases where outcomes are closely tied to stage and surgical margins [3,4].

Postoperative follow-up is essential for all urachal neoplasms, given the potential for recurrence or residual symptoms. This case illustrates the importance of addressing lower urinary tract dysfunction and scrotal pain, which developed postoperatively. A multidisciplinary approach involving urology, oncology, and pathology is critical for managing these rare but clinically significant tumors.

Table 1 provides a detailed comparison of the histological, immunophenotypic, molecular, and clinical features of urachal mucinous neoplasms, emphasizing their distinct management and prognostic considerations.

**Table 1:** Comparative features of urachal mucinous neoplasms. MCTLMP: Mucinous cystic tumor of low malignant potential

Feature	Cystadenoma	MCTLMP	Adenocarcinoma
Histology	Simple mucinous epithelium, minimal atypia	Papillary/villous architecture, mild-to-moderate atypia, no stromal invasion	Marked cytologic atypia, stromal invasion, complex architecture
Immunophenotype (IHC)	Limited data	CK20+, CDX2+ (intestinal differentiation), variable GATA3 and CK7 expression	CK20+, CDX2+, aberrant patterns of GATA3 (loss), CK7 (variable/lost), p53 (mutant), β-catenin (nuclear), and AMACR (strong expression)
Molecular Insights	Rarely characterized	KRAS mutations implicated, potential role in prognosis	KRAS and BRAF mutations linked to tumor progression
Clinical Presentation	Often asymptomatic, discovered incidentally	Dysuria, suprapubic discomfort, mucinuria	Hematuria, systemic symptoms, advanced disease presentation
Treatment	Curative with simple excision	Complete excision, including urachal remnant	Radical resection with lymphadenectomy, possible chemotherapy
Prognosis & Follow-Up	Excellent, negligible recurrence	Favorable with appropriate surgical management	Poor due to recurrence and metastasis risks

## Conclusions

Mucinous cystic tumors of the urachus, including MCTLMPs, represent a rare but clinically significant entity spanning benign cystadenomas to invasive adenocarcinomas. Accurate histopathological evaluation, supported by immunohistochemical and molecular studies, is crucial to distinguish these lesions and guide appropriate management. This case contributes to the growing understanding of MCTLMP, a rare borderline lesion in the spectrum of urachal mucinous neoplasms. The favorable outcome following complete excision underscores the importance of accurate diagnosis, thorough histopathological evaluation, and appropriate surgical management. Complete surgical excision remains the cornerstone of treatment for both benign and borderline lesions, effectively preventing progression and recurrence. In invasive cases, early detection, radical resection, and a multidisciplinary approach are paramount for optimizing outcomes, particularly in the absence of standardized systemic therapies.

Future research should aim to establish consensus diagnostic criteria, explore molecular pathways such as KRAS mutations, and incorporate biomarkers like CEA and CA19-9 into long-term surveillance strategies to refine prognostication and therapeutic interventions. As molecular profiling evolves, it may provide insights into targeted therapies and improve clinical outcomes for these rare tumors. Cases such as the one presented highlight the need for heightened awareness, comprehensive histopathological workup, and vigilant long-term follow-up to enhance patient care and management.

## References

[1] Reek C, Graefen M, Erbersdobler A, Haese A (2000). Mucinous adenocarcinoma of the urachus. Case report and review of the literature (Article in German). Urologe A.

[2] Eble JN, Hull MT, Rowland RG, Hostetter M (1986). Villous adenoma of the urachus with mucusuria: a light and electron microscopic study. J Urol.

[3] Gopalan A, Sharp DS, Fine SW, Tickoo SK, Herr HW, Reuter VE, Olgac S (2009). Urachal carcinoma: a clinicopathologic analysis of 24 cases with outcome correlation. Am J Surg Pathol.

[4] Bruins HM, Visser O, Ploeg M, Hulsbergen-van de Kaa CA, Kiemeney LA, Witjes JA (2012). The clinical epidemiology of urachal carcinoma: results of a large, population based study. J Urol.

[5] Choi JW, Lee JH, Kim YS (2012). Urachal mucinous tumor of uncertain malignant potential: a case report. Korean J Pathol.

[6] Amin MB, Smith SC, Eble JN, Rao P, Choi WW, Tamboli P, Young RH (2014). Glandular neoplasms of the urachus: a report of 55 cases emphasizing mucinous cystic tumors with proposed classification. Am J Surg Pathol.

[7] Dhillon J, Liang Y, Kamat AM, Siefker-Radtke A, Dinney CP, Czerniak B, Guo CC (2015). Urachal carcinoma: a pathologic and clinical study of 46 cases. Hum Pathol.

[8] Paner GP, Lopez-Beltran A, Sirohi D, Amin MB (2016). Updates in the pathologic diagnosis and classification of epithelial neoplasms of urachal origin. Adv Anat Pathol.

[9] Jayakumar S, Darlington D (2020). Acute presentation of urachal cyst: a case report. Cureus.

[10] Agrawal AK, Bobiński P, Grzebieniak Z (2014). Pseudomyxoma peritonei originating from urachus-case report and review of the literature. Curr Oncol.

[11] Reis H, Krafft U, Niedworok C (2018). Biomarkers in urachal cancer and adenocarcinomas in the bladder: a comprehensive review supplemented by own data. Dis Markers.

[12] Sugiyama K, Ito N (2009). Mucinous cystadenocarcinoma of the urachus associated with pseudomyxoma peritonei with emphasis on MR findings. Magn Reson Med Sci.

[13] Garcia JP, Sampaio R, Peixoto C (2017). Urachal tumor: a case report of an extremely rare carcinoma. Case Rep Pathol.

[14] Wang D, Sule N (2019). Mucinous cystadenoma of the urachus and review of current classification of urachal mucinous cystic neoplasms. Arch Pathol Lab Med.

[15] Van Allen J (2021). A rare case of urachal adenocarcinoma with bone marrow metastasis. BMJ Case Rep.

